# Dissemination of a quality improvement intervention to reduce early term elective deliveries and improve birth registry accuracy at scale in Ohio

**DOI:** 10.1186/1748-5908-10-S1-A2

**Published:** 2015-08-14

**Authors:** Heather C Kaplan, Colleen Mangeot, Susan N Sherman, Charlena Cleveland, Sandra Fuller, Beth E White, Susan Ford, Michael A Krew, Michael P Marcotte, Jay D Iams, Jennifer Bailit, Jo M Bouchard, Kelly Friar, Eileen King, Carole Lannon

**Affiliations:** Perinatal Institute, Cincinnati Children's Hospital Medical Center, Cincinnati, OH 45229 USA; James M. Anderson Center for Health Systems Excellence, Cincinnati Children's Hospital Medical Center, Cincinnati, OH 45229 USA; Division of Biostatistics and Epidemiology, Cincinnati Children's Hospital Medical Center, Cincinnati, OH 45229 USA; SNS Research, Cincinnati, OH 45249 USA; Ohio Beacon Council and The Ohio Colleges of Medicine Government Resource Center, Columbus, OH 43210 USA; Department of Obstetrics & Gynecology, Division of Maternal Fetal Medicine, Aultman Hospital, Canton, OH 44710 USA; Department of Obstetrics & Gynecology, Division of Maternal Fetal Medicine, Good Samaritan Hospital, Cincinnati, OH 45220 USA; Department of Obstetrics & Gynecology, The Ohio State University Wexner Medical Center, Columbus, OH 43210 USA; Division of Maternal-Fetal Medicine, Department of Obstetrics and Gynecology, Metrohealth Medical Center, Cleveland, OH 44109 USA; Ohio Department of Health, Bureau of Maternal and Child Health, Columbus, OH 43215 USA; Bureau of Vital Statistics, Ohio Department of Health, Columbus, OH 43215 USA

## Background

After completing a successful quality improvement (QI) initiative to decrease non-medically indicated scheduled births <39 weeks in 20 large maternity hospitals, the Ohio Perinatal Quality Collaborative (OPQC) aimed to expand this work across Ohio. Our objective was to evaluate the effectiveness of the QI intervention used by OPQC to rapidly spread the 39 week initiative.

## Methods

Participating hospitals were exposed to an 8-month QI intervention to reduce non-medically indicated scheduled deliveries <39 weeks and reflect those improvements in accurate state vital statistics data. The intervention was implemented using a step-wedge design with hospitals divided into three balanced waves. Effectiveness was assessed using interrupted time-series analysis. Interviews with a sample of participants and project call recordings were analyzed using qualitative methods to understand implementation.

## Results

Seventy of the 72 (97%) eligible hospitals agreed to participate. Hospitals actively participated as evidenced by 95.7% attending the in-person learning session and 80% attending at least three of four monthly group calls. Based on birth registry data, rates of non-medically indicated inductions <39 weeks declined in all waves concurrent with the start of the intervention. Changes related to reducing deliveries <39 weeks were variably implemented--some hospitals described extensive changes as a result of their participation while others who had previously begun work in this area described either small changes or no changes. Participants implemented multiple changes to improve birth registry accuracy.

## Conclusions

We describe a QI intervention enabling state-wide spread of effective change strategies over 12 months. This intervention could support a model where a network of innovator hospitals develops and tests change strategies and then rapidly spreads successful approaches to a broader population. Such a model has the potential to advance dissemination and implementation science.

## Primary funding sources

AHRQ U19HS02114, CDC 31-90000-553005-130488, and Ohio Government Resource Center.

